# Validation of the Chinese Cultural Tightness–Looseness Scale and General Tightness–Looseness Scale

**DOI:** 10.3389/fpsyg.2023.1131868

**Published:** 2023-04-18

**Authors:** Jie Leng, Hang Ma, Xiaojun Lv, Ping Hu

**Affiliations:** Department of Psychology, Renmin University of China, Beijing, China

**Keywords:** cultural tightness–looseness, general tightness–looseness, latent profile analysis, well-being, validation

## Abstract

**Introduction:**

This study aims to revise the Cultural Tightness–Looseness Scale (CTLS) and General Tightness–Looseness Scale (GTLS), and explore the group heterogeneity of tightness–looseness perception in Chinese populations.

**Methods:**

Sample 1 (*n* = 2,388) was used for item analysis and exploratory factor analysis, and sample 2 (*n* = 2,385) was used for confirmatory factor analysis and latent profile analysis. Sample 3 (*n* = 512) was used for the reliability test and criterion validity test, among which 162 participants were used for the test–retest reliability examination after a four-week interval. Measurements included the CTLS, GTLS, International Personality Item Pool, Personal Need for Structure Scale, and Campbell Index of Well-Being.

**Results:**

The revised CTLS contained four items and retained a single-dimensional structure. The revised GTLS consisted of eight items divided into two dimensions: Compliance with Norms and Social Sanctions. Latent profile analysis extracted two profiles on both CTLS and GTLS scores, indicating that the sample can be divided into two subgroups: high and low perception of tightness.

**Discussion:**

The Chinese versions of the CTLS and GTLS can be used as valid and reliable measures of tightness–looseness perception in a Chinese population.

## 1. Introduction

Social norms, defined as general unwritten standards of behavior, are vital to both individuals and society (Gelfand et al., 2006; Hawkins et al., 2019). However, there may be national, regional, or social differences in the strength of social norms and the degree of sanctions for violating them. These differences describe cultural tightness–looseness (Gelfand et al., 2006), which is influenced by ecological and historical factors, such as population density and the occurrence of epidemics (Chan, 1996; Gelfand et al., 2011). When facing collective threats, tighter cultures hold an evolutionary advantage–for example, in a study of nation-level cultures, tighter cultures were associated with lower mortality rates by epidemics, even when controlling for collectivism/individualism (Gelfand et al., 2021).

Additionally, research demonstrates that cultural tightness and looseness can affect individuals’ personalities, emotions, and behaviors. For example, individuals in tight cultures tend to be more conscientious and have higher self-monitoring and self-control abilities which help them in adjusting their behavior in real-time to conform to social norms (Gelfand et al., 2011). Cultural tightness is also associated with mental health; for instance, individuals in tighter cultures are more likely to express positive emotions and experience higher life satisfaction (Liu et al., 2018; Chua et al., 2019) in the Chinese context, while tighter culture may lead to lower happiness (Harrington and Gelfand, 2014) in American. In addition, a tight culture is associated with higher interdependent self-concepts (Carpenter, 2000), and may encourage more charitable giving (Siemens et al., 2020). However, tighter culture could reduce innovation (Chua et al., 2019), decrease a sense of individual personal control (Ma et al., 2022), and grow more prejudice based on skin color, sexuality and so on (Jackson et al., 2019). Cultural tightness–looseness also plays important roles in other fields of study, such as management (e.g., Gedik and Ozbek, 2020) and advertising (e.g., Youn et al., 2019).

Previous work has measured cultural tightness and looseness in various ways. Harrington and Gelfand (2014) synthesized objective indicators such as the severity of punishment and degree of religious piety as a measure of the regions’ cultural tightness–looseness, while Jackson et al. (2019) calculated the frequency of words associated with cultural tightness–looseness. Developed by Gelfand et al. (2011), the Cultural Tightness–Looseness Scale (CTLS) measures the perceived cultural tightness at a personal level (Marcus et al., 2022). In addition, predecessors composed a provincial (Chua et al., 2019) and national (Mandel and Realo, 2015) level of cultural tightness–looseness by a large sample using CTLS. The CTLS is a single-factor structure measurement that assesses the degree of clarity and effective implementation of social norms through six items and is widely applied in surveys related to cultural tightness–looseness. Nevertheless, the reliability and validity of the CTLS have been seriously challenged when tested in different cultural contexts (Treviño et al., 2021). Oh (2022) found that the factor loading of a reversed-scoring item in the CTLS was low when used with a Korean population, and that after removing this item, the scale reliability was only 0.67. In another study assessing cultural tightness–looseness amongst Chinese participants, Liu et al. (2022) reported a Cronbach’s alpha of 0.68 for two items of the CTLS. This demonstrates the need to revise CLTS and modify it to be suitable for measuring the cultural tightness–looseness in China.

The Cultural tightness–looseness theory (Gelfand et al., 2011) posits that the strength of social norms, and the tolerance of deviant behavior affect psychological adaptation through the structure of people’s daily life situations and how constraining the situation is. Therefore, individuals may experience situational tightness differently in their daily life because of the difference in cultural tightness. In recent years, the process of urbanization in China has promoted the diversification of people’s lifestyles (He and Qian, 2017). Although within the same macro-cultural context, individual microsystems may be diverse nowadays. Importantly, as ecological systems theory suggests, the microsystem is the immediate environment for children’s development (Bronfenbrenner, 1992). Thus, the degree to which individuals perceive the tightness of their general lives may have a more direct effect on their behaviors. Harrington (2017) developed the General Tightness–Looseness Scale (GTLS) and Childhood Home-Life Tightness–Looseness Scale, among others, to assess perceived tightness–looseness in specific domains. He found that tightness perceived in general life is positively associated with that perceived in other specific domains, and it is also positively related to an individual’s conscientiousness and self-monitoring. Since the inception of the GTLS, it has not been applied to measure Chinese people’s perception of the general tightness–looseness of their life. Given the differences in people’s perception of general life tightness and its more direct effects on individual behavior, this study also aims to adapt the GTLS in the Chinese setting.

In addition, the level at which people perceive cultural tightness varies despite the same macro-cultural context (Harrington and Gelfand, 2014; Chua et al., 2019). Therefore, the revised CTLS and GTLS should be able to effectively distinguish between individuals with different degrees of perceived cultural tightness and looseness. Latent Profile Analysis (LPA) is an individual-centered method that can identify different subgroups contained in a population (Howard and Hoffman, 2018). The LPA can also be used to evaluate the construct validity of scales (Davis et al., 2015; Baek et al., 2022). Paschke et al. (2020) applied LPA to analyze the newly compiled Gaming Disorder Scale for Adolescents and found that adolescents with different levels of gaming disorders could be effectively distinguished, indicating that the scale had good discrimination ability. LPA will be used to examine construct validity in this study and explore the pattern of perceived cultural differences in tightness–looseness amongst the Chinese population.

## 2. Materials and methods

### 2.1. Participants and procedure

Two rounds of data collection were conducted in this study. The first round was based on a psychological and health behavior status survey program for college freshmen. The research assistants gave the participants links to an online survey and reminded them to complete it while waiting in line for a physical examination. The response rate was 75.51%. The second round of test was conducted 4 weeks later through the online platform (Wenjuanwang). The participants volunteered to participate and were paid 5 RMB remuneration. The response rate of the survey was 99.03%. Of those, 31.64% took part in the first round of testing. This study was approved by the Ethics Committee of the Department of Psychology, Renmin University of China.

#### 2.1.1. Sample 1 and sample 2

A total of 4,773 Chinese teenagers and adults volunteered to participate in the first round. According to Raylu and Oei (2004), the samples were randomly divided into two groups, namely, sample 1 and sample 2.

Sample 1 was used for item analysis and exploratory factor analysis (EFA). A total of 2,388 participants were included of which, 1,591 were females, ranging from 14 to 46 years of age, with an average age of 21.76 years (*SD* = 4.26). Sample 2 was used for CFA and LPA. A total of 2,385 people were included of which, 1,508 were females, ranging from 15 to 45 years old, with an average age of 21.70 years (*SD* = 4.23).

Given that Sample 1 (*χ*^2^ = 264.00, *df* = 1, *p* < 0.001) and Sample 2 (*χ*^2^ = 166.94, *df* = 1, *p* < 0.001) consisted of more women than men, we tested whether there were significant gender differences in the revised CTLS and GTLS scores. In Sample 1, there were significant gender differences in CTLS [*t*(2386) = −2.694, *p* < 0.01, cohen’s *d* = −0.11] and GTLS [*t*(2386) =3.104, *p* < 0.01, cohen’s *d* = 0.13] scores. In Sample 2, only a significant gender difference in CTLS score [*t*(2383) = −5.83, *p* < 0.001, cohen’s *d* = −0.24] was found. However, cohen’s *d* values for these tests were very small.

#### 2.1.2. Sample 3

The data for sample 3 came from the second round of data collection and was used for the reliability test and criterion validity test. This sample included 512 participants (*N*_female_ = 252, *M*_age_ = 20.63, *SD*_age_ = 2.69). Among them, 162 (*N*_female_ = 115, *M*_age_ = 19.79, *SD*_age_ = 3.04) participants had completed the first round measurement, and they were used to investigate the retest reliability at an interval of 4 weeks.

### 2.2. Materials and measures

#### 2.2.1. CTLS, GTLS and translation process

##### 2.2.1.1. Cultural tightness–looseness scale

The English version of the CTLS (Gelfand et al., 2011) is a single-dimension scale consisting of six items, of which the fourth item is reverse-scored. A six-point Likert scale (1 = strongly disagree, 6 = strongly agree) is used to calculate the total score. The higher the total score, the tighter the perception of national culture (Gelfand et al., 2011). An example of an item in the scale is “In this country, there are very clear expectations for how people should act in most situations.” The Chinese translation of CTLS refers to the translation by Chua et al. (2019) because it is accurate and authentic. The revised version of the Chinese scale is included in the Supplementary material.

##### 2.2.1.2. General tightness–looseness scale

The English version of the 13-item GTLS (Harrington, 2017) has a single-factor structure, of which items 4, 12, and 13 are reverse-scored. Participants used a six-point Likert scale (1 = strongly disagree, 6 = strongly agree) to rate their agreement with each statement, with a higher total score suggesting a tighter perception of their overall life (Harrington, 2017). A sample item is “In my life, if I act in an inappropriate way, others will strongly disapprove.” GTLS was translated into Chinese and then translated back by two psychology researchers. After discussion and modification, the initial Chinese version of the GTLS scale was determined.

#### 2.2.2. Criterion validity measurements

Gelfand et al. (2011) argued that cultural tightness can influence people’s dutifulness, impulse self-control, and individual structural needs through daily situational constraints. Thus, these variables were used as criterions in this revision. Furthermore, Chua et al. (2019) found a positive relationship between cultural tightness and well-being in the Chinese cultural context; therefore, well-being was also used as a criteria variable.

##### 2.2.2.1. International personality item pool

The 120-item International Personality Item Pool (IPIP; Johnson, 2014) measures the Big Five personality traits. This study utilized the two subscales measuring dutifulness and impulse control from the Chinese version of the IPIP (Ge, 2016). These subscales comprise a total of eight items, with sample items including statements like “keeping your promises” and “controlling your desires.” Participants indicate how consistent these statements are for them on a 5-point Likert scale (1 = strongly inconsistent, 5 = strongly consistent). Higher scores indicate higher personal dutifulness and greater impulse control. In this study, Cronbach’s α of the dutifulness subscale was 0.67, and that of the impulse control subscale was 0.68.

##### 2.2.2.2. Personal need for structure scale

The Personal Need for Structure Scale (PNS) assesses the extent to which people tend to construct their worlds in simple and unambiguous ways (Neuberg and Newsom, 1993). The Chinese version of the PNS contains a total of 11 items (Chen et al., 2008), scored from 1 (strongly disagree) to 6 (strongly agree), with items such as “I enjoy having a clear and structured mode of life.” A larger score indicates a greater personal need for structure. The Cronbach’s α for the PNS was 0.75.

##### 2.2.2.3. The campbell index of well-being

The Chinese version of the Campbell Index of Well-being (Campbell, 1976; Wang et al., 1999) is widely used to measure the well-being of Chinese people (e.g., Liu et al., 2019), with two subsections: index of general affect, and overall life satisfaction item. The eight-item index of general affect asks participants to rate how often they experience a variety of emotions on a scale from 1 (very dissatisfied) to 7 (very satisfied). The overall life satisfaction item is composed of a single item that asks “How satisfied are you with your life as a whole?” and scored on a 7-point Likert scale (1 = very dissatisfied, 7 = very satisfied). Index of Well-Being = 1.1 * (overall life satisfaction item) + 1.0 * (Index of General Affect). Cronbach’s α for the total scale was 0.92, and that of the index of general affect was 0.91.

## 3. Results

We used SPSS 22.0 for item analysis, EFA, and reliability and validity tests. Confirmatory factor analysis and LPA were conducted using Mplus 7.0.

### 3.1. Item analysis

Sample 1 was used for item analysis. The participants were divided according to their total scores on the CTLS, and scores in the upper 27% and lower 27% were selected for measuring *t-*test (Yildiz et al., 2009). The same analytical method was applied with GTLS. Correlation between each item and total scores was calculated (e.g., Akin et al., 2013), as shown in Table 1. The score for the fourth CTLS item in the upper 27% group (1.52 ± 1.08) was significantly lower than that in the lower 27% group (2.56 ± 1.05), and the item-total correlation was significantly negative, indicating that it made a negative contribution to the total score and could not effectively distinguish between high and low CTLS score; thus, this item was deleted. Similarly, items 4, 12 and 13 of the GTLS were excluded.

**Table 1:** *t*-text and item-total correlation (*N* = 2,388).

	*t*	Item- total correlation
*CTLS (In this country……)*		
CTLS1 There are many social norms that people are supposed to abide by in this country.	−26.33^***^	0.53^***^
CTLS2 There are very clear expectations for how people should act in most situations.	−44.22^***^	0.77^***^
CTLS3 People agree upon what behaviors are appropriate versus inappropriate in most situations this country.	−44.44^***^	0.78^***^
CTLS4 People in this country have a great deal of freedom in deciding how they want to behave in most situations.	19.02^***^	−0.31^***^
CTLS5 If someone acts in an inappropriate way, others will strongly disapprove.	−41.50^***^	0.70^***^
CTLS6 People in this country almost always comply with social norms.	−44.81^***^	0.75^***^
*GTLS (In my life……)*		
GTLS1 There are many rules that I am supposed to follow in my life.	−25.48^***^	0.51^***^
GTLS2 There are very clear expectations for how I should act in most situation.	−23.99^***^	0.49^***^
GTLS3 It is clear what behaviors are appropriate versus inappropriate in my life.	−23.75^***^	0.50^***^
GTLS4 I have a great deal of freedom in deciding how I want to behave in most situations.	10.43^***^	−0.22^***^
GTLS5 If I act in an inappropriate way, others will strongly disapprove.	−38.57^***^	0.66^***^
GTLS6 I almost always follow the rules.	−36.71^***^	0.66^***^
GTLS7 People closely monitor what I do.	−36.35^***^	0.63^***^
GTLS8 There are strong punishments if I do not follow the rules.	−47.25^***^	0.74^***^
GTLS9 My life is very structured. I know what I should and should not be doing.	−22.93^***^	0.46^***^
GTLS10 There is a right way and a wrong way to do things.	−40.51^***^	0.68^***^
GTLS11 There is a rule or a proper procedure for most things.	−31.96^***^	0.63^***^
GTLS12 I often have a choice in deciding what I want to do in my life.	6.58^***^	−0.13^***^
GTLS13 I often have a choice in deciding when I want to do something in my life.	6.33^***^	−0.13^***^

^***^p < 0.001.

### 3.2. Construct validity

#### 3.2.1. Exploratory factor analysis

##### 3.2.1.1. Cultural tightness–looseness scale

Sample 1 was used to conduct EFAs for the CTLS and GTLS. We first ran an EFA with the five items from the original CTLS after removing item 4 based on the item analysis above. EFA results indicated that the KMO value was 0.78, and Bartlett’s test of sphericity was significant (*χ*^2^ = 4516.57, *df* = 10, *p* < 0.001). Using Principal Component Analysis and Varimax Rotation, only one factor with an eigenvalue greater than 1.00 was extracted, which explained 57.79% of the total variance. Based on the criteria of commonality greater than 0.3 and factor load greater than 0.4 (Du et al., 2022), item 1 of the CTLS (commonality = 0.26) was excluded. We then ran a second EFA with four items (i.e., after removing item 1). Results showed the KMO value was 0.77, and Bartlett’s spherical test was significant (*χ*^2^ = 4106.39, *df* = 6, *p* < 0.001). The single factor with the remaining four items explained 67.55% of the total variance. Commonalities and factor loadings are presented in Table 2 (*N* = 2,388).

**Table 2:** Exploratory factor analysis (*N* = 2,388).

	Communalities	Factor 1	Factor 2
*CTLS*			
CTLS1	0.26	0.51	
CTLS2	0.70(0.71)	0.84(0.84)	
CTLS3	0.74(0.76)	0.86(0.87)	
CTLS5	0.65(0.54)	0.74(0.73)	
CTLS6	0.54(0.69)	0.80(0.83)	
*GTLS*			
GTLS1	0.57(0.56)	0.74(0.73)	0.16(0.16)
GTLS2	0.71(0.73)	0.83(0.84)	0.13(0.14)
GTLS3	0.68(0.70)	0.81(0.82)	0.15(0.16)
GTLS5	0.47(0.52)	0.31(0.33)	0.61(0.64)
GTLS6	0.52	0.51	0.51
GTLS7	0.55(0.58)	0.08(0.10)	0.73(0.75)
GTLS8	0.64(0.65)	0.13(0.14)	0.79(0.80)
GTLS9	0.52(0.53)	0.68(0.69)	0.26(0.24)
GTLS10	0.57(0.57)	0.15(0.17)	0.74(0.74)
GTLS11	0.51	0.46	0.55

The communalities and factor loadings outside brackets represent the result of the first EFA. While the values in brackets represent the EFA result after deleting items (item 1 in CTLS and items 6, and 11 in GTLS). The factor loadings of the CTLS were those before factor rotation (since only one factor was extracted) and the factor load of the GTLS was those after factor rotation. Compliance with norms dimension (factor 1) includes GTLS1, GTLS2, GTLS3, and GTLS9. Social sanctions dimension (factor 2) includes GTLS5, GTLS7, GTLS8, and GTLS10.

##### 3.2.1.2. General tightness–looseness scale

We first ran an EFA with the ten items from the original GTLS after removing items 4, 12 and 13 based on the item analysis. EFA results demonstrated that the KMO value was 0.80, and Bartlett’s test of sphericity was significant (*χ*^2^ = 8388.92, *df* = 45, *p* < 0.001). Principal Component Analysis and Varimax Orthogonal Rotation were used to extract the factors and rotation factors, respectively. Two factors were extracted. However, only 57.33% of the cumulative variance contribution was explained. Among the items, the factor loadings for item 6 in Factor 1 and Factor 2 were both greater than 0.4, and the difference in absolute values was less than 0.3. This item was therefore deleted according to the suggestion of Du et al. (2022), along with item 11. After removing items 6 and 11, the EFA with eight items showed that the KMO value became 0.84, and Battlett’s spherical test indicated a significant result (*χ*^2^ = 5828.65, *df* = 28, *p* < 0.001). Two factors were extracted, which explained 60.53% of the total variance (factor 1: 32.02%, factor 2: 28.51%). Factor 1 was named “Compliance with Norms,” which measured the clarity of the social norms and the extent to which people should follow them in general life and factor 2 was named “Social Sanctions,” which measured the degree to which people were sanctioned for violating norms. Table 2 presents the EFA results for all items.

#### 3.2.2. Confirmatory factor analysis

Sample 2 was used to conduct CFA for both CTLS and GTLS. A Kolmogorov–Smirnov test demonstrated that the scores of each item in the CTLS and GTLS were not normally distributed. CFA was conducted using the robust maximum likelihood (MLR) method (Suh, 2015). The results suggest that the revised CTLS fits a single-factor model well (*χ*^2^ = 37.41, *df* = 2, CFI = 0.98, TLI = 0.93, RMSEA = 0.086, SRMR = 0.03) (Gómez-López et al., 2020).

Additionally, the revised two-dimensional GTLS had a good fit to the data (*χ*^2^ = 208.22, *df* = 19, CFI = 0.96, TLI = 0.93, RMSEA = 0.07, SRMR = 0.04), whereas the one-factor model did not indicate satisfactory construct validity (*χ*^2^ = 1157.42, *df* = 20, CFI = 0.73, TLI = 0.63, RMSEA = 0.15, SRMR = 0.09). Therefore, consistent with the EFA findings, a two-factor structure was more suitable for the revised GTLS than a one-factor structure.

#### 3.2.3. Latent profile analysis

LPAs for the CTLS items and the GTLS items were conducted separately using data from Sample 2. Commonly used evaluation indices of LPA model solutions include AIC, BIC, and aBIC, with smaller values indicating better model fits. Entropy generally needs to be larger than 0.8, with entropy closer to 1 indicating a lower classification uncertainty in the model (Tein et al., 2013). In addition, the Lo–Mendell–Rubin (LMR) test and the bootstrap likelihood ratio test (BLRT) were used to compare a k-class model with a k-1 class model, and significant results of the LMR and BLRT indicate that the current model has a significant better fit than that with one less class (Davis et al., 2015). It should be noted that profiles with less than 5% of the sample size may be spurious – when the number of people in a certain category is very small, the classification may be problematic (Tein et al., 2013; Ferguson et al., 2020).

**Figure 1:**
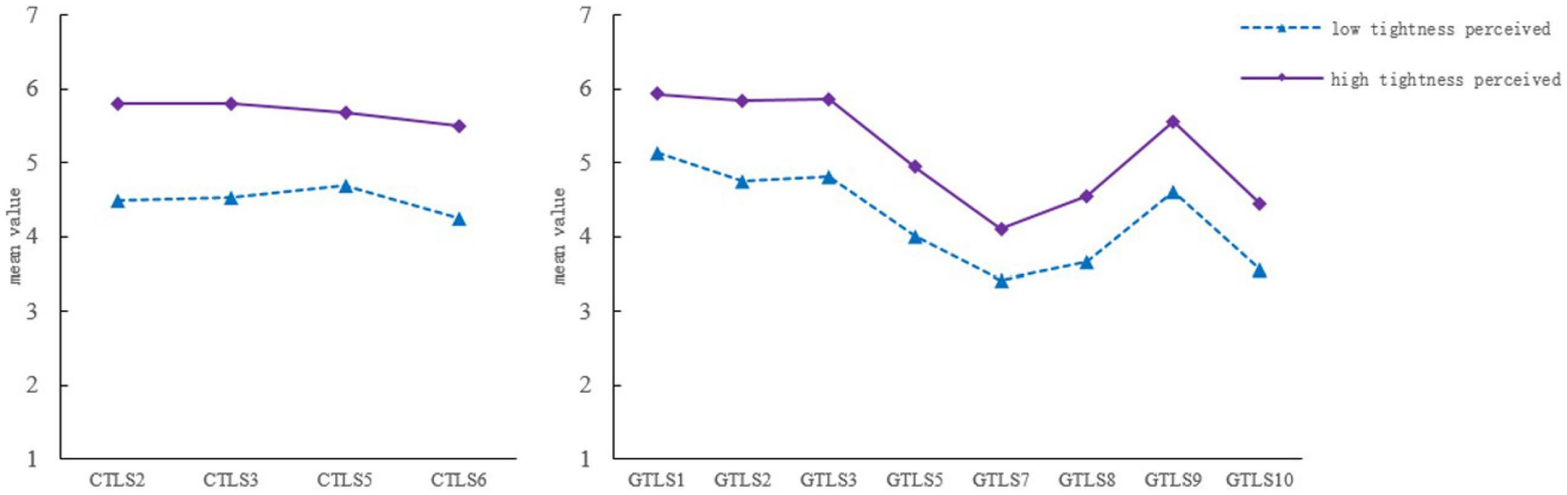
LPA of perceived national cultural tightness and general life tightness.

The three- and four-class models were excluded from consideration because they yielded a non-significant LMR (see Table 3). The two-class model yielded lower AIC, BIC, and aBIC values than the one-class model. Additionally, the entropy of the two-class model reached the criterion of 0.8. Based on these results, the two-class model was the most appropriate; in other words, participants were divided into two subgroups based on their CTLS and GTLS scores. The classification results and the mean scores of each item are shown in Figure 1.

**Table 3:** Fit statistics and class probability for LPA.

Model	AIC	BIC	aBIC	Entropy	LMR	BLRT	Class probability (%)
*CTLS*							
1-class	24523.63	24569.84	24544.43				100
2-class	21334.59	21409.69	21368.38	0.80	*p* < 0.001	*p* < 0.001	38.41/61.59
3-class	19243.11	19347.10	19289.91	0.99	*p* > 0.05	*p* < 0.001	13.92/32.70/53.38
4-class	15249.51	15382.38	15309.30	1	*p* > 0.05	*p* < 0.001	10.27/32.66/53.42/3.65
*GTLS*							
1-class	51493.48	51585.91	51535.07				100
2-class	47281.65	47426.07	47346.64	0.88	*p* < 0.001	*p* < 0.001	37.23/62.77
3-class	44291.83	44488.24	44380.22	1	*p* > 0.05	*p* < 0.001	9.31/58.03/32.66
4-class	42857.52	43105.93	42969.31	0.93	*p* > 0.05	*p* < 0.001	34.80/34.30/20.59/10.31

AIC, Akaike information criterion; BIC, Bayesian information criteria; aBIC, sample-adjusted BIC; LMR, Lo–Mendell–Rubin test; BLRT, Bootstrap likelihood ratio test; Entropy is an indicator of classification uncertainty; category probability reveals the proportion of the people contained in a category.

The two categories were named as the low-tightness perception group and the high-tightness perception group, and the scores on each item of the high-tightness perception group were higher than that of the low-tightness perception group. We treated the LPA class as a classification variable and conduct further analysis. The CTLS total scores of the two groups were compared, and a significant difference [*t*(1317.23) = −61.19, *p* < 0.001, Cohen’s *d* = −2.72] was found. More women perceived the national culture tighter (*χ*^2^ = 18.43, *df* = 1, *p* < 0.001), while the age difference between the two groups was not significant. The score of GTLS was significantly different between the high and low tightness perception groups [*t*(2017.33) = −46.65, *p* < 0.001, Cohen’s *d* = −1.95], while the gender distribution (*χ*^2^ = 2.95, *df* = 1, *p* > 0.05) of the two groups and age difference [*t*(2046.09) = −1.82, *p* > 0.05] were not significant.

### 3.3. Reliability

#### 3.3.1. Internal consistency reliability

Sample 3 was used to test the internal consistency and reliability of the revised scale. The Cronbach’s α of CTLS was 0.78, and the Cronbach’s α of GTLS was 0.82 (compliance with norms: Cronbach’s *α* = 0.74; social sanctions: Cronbach’s *α* = 0.79).

#### 3.3.2. Retest reliability

Results showed that all Pearson’s correlation and intraclass correlation coefficients were significant (*p* < 0.001): CTLS (*r* = 0.47, ICC = 0.64), GTLS (*r* = 0.52, ICC = 0.68), compliance with norms (*r* = 0.58, ICC = 0.72), and social sanctions (*r* = 0.48, ICC = 0.64).

### 3.4. Criterion validity

The results of the criterion validity are shown in Table 4 (*N* = 512). The CTLS was positively related to the GTLS and its two dimensions. Both the CTLS and GTLS were positively associated with dutifulness, impulse control, and personal need for structure and well-being. All the above correlations were statistically significant. In addition, the structural equation modeling (SEM) method was used to test criterion validity, with the multiple criterion variables included simultaneously in the SEM. Results showed that both CTLS (*χ*^2^/df = 2.33, CFI = 0.91, TLI = 0.90, RMSEA = 0.05) and GTLS (*χ*^2^/df = 2.39, CFI = 0.92, TLI = 0.90, RMSEA = 0.05) model fitted the data well. In addition, all the predicted paths were significant (see Table 5).

**Table 4:** Criterion validity (*N* = 512).

Variables	*M ± SD*	1	2	3	4	5	6	7	8	9
1. CTLS	19.69 ± 2.98									
2. GTLS	37.53 ± 5.76	0.66^***^								
3. Compliance with norms	20.11 ± 2.76	0.64^***^	0.81^***^							
4. Social sanctions	17.42 ± 3.88	0.52^***^	0.91^***^	0.49^***^						
5. Dutifulness	16.25 ± 2.79	0.32^***^	0.31^***^	0.43^***^	0.15^**^					
6.Impulse control	14.65 ± 2.84	0.33^***^	0.36^***^	0.40^***^	0.25^***^	0.47^***^				
7. Personal need for structure	46.12 ± 7.55	0.22^***^	0.21^***^	0.28^***^	0.12^**^	0.32^***^	0.16^***^			
8. Well-Being	45.83 ± 9.34	0.41^***^	0.41^***^	0.41^***^	0.33^***^	0.28^***^	0.45^***^	0.06		
9. Index of General Affect	40.20 ± 8.30	0.41^***^	0.42^***^	0.41^***^	0.34^***^	0.28^***^	0.45^***^	0.05	0.99^***^	
10. Overall Life satisfaction	5.12 ± 1.25	0.33^***^	0.27^***^	0.31^***^	0.19^***^	0.22^***^	0.32^***^	0.05	0.79^***^	0.72^***^

^**^p < 0.01, ^***^p < 0.001.

**Table 5:** SEM for criterion validity test (*N* = 512).

Path	Estimate	Path	Estimate
CTLS		GTLS	
Dutifulness	0.24^***^	Dutifulness	0.13^***^
Impulse control	0.36^***^	Impulse control	0.12^***^
Personal need for structure	0.34^***^	Personal need for structure	0.12^***^
Well-Being	0.65^***^	Well-Being	0.19^***^

^***^p < 0.001.

## 4. Discussion

This study revised the CTLS and GTLS in the Chinese context. Firstly, item analysis showed that some items such as CTLS4 could not distinguish between high and low levels of perceived tightness. In contemporary China, freedom has become a core social value and is constrained by collective interests (Gow, 2017), people may perceive both being constrained by social norms and greater freedom. Therefore, CTLS4 is not good at distinguishing the degree of perceived tightness–looseness, as well as items 4, 12 and 13 of the GTLS. In addition, as suggested by Baumgartner et al. (2018), the core concept of the reverse scoring item is contrary to those of other items in the scale, which may reduce the reliability and misleading factor structure of the scale. Therefore, these items were excluded from the revised scales.

The revised Chinese version of the CTLS is structurally consistent with the version by Gelfand et al. (2011). It remains a single-factor structure that assesses how clearly and compliantly people perceive social norms at the country level. In addition, we adapted the GTLS to measure tightness and looseness in general life. This revised GTLS extracted two dimensions named as “compliance with norms” and “social sanctions.” The definition of cultural tightness–looseness includes two pivotal aspects: the intensity of social norms and the degree of sanctions within society (Gelfand et al., 2006). Therefore, although the revised GTLS differs from the one-dimensional structure proposed by Harrington (2017), it is more consistent with the definition of cultural tightness–looseness. Besides, this study found a significantly positive relationship between CTLS and GTLS. Gelfand et al. (2011) found that the national-level strength of societal norms and tolerance of deviant behavior will influence the degree of everyday situational constraint, while this study discovered the individual level of perceived cultural tightness–looseness was associated with those perceived in general life. These findings may enrich the cultural tightness–looseness theory (Gelfand et al., 2011) in that macro-culture affects the constraint of individuals’ daily living situations both at national and personal perceived levels.

Furthermore, the results of LPA demonstrate that participants could be grouped into two heterogeneous sub-groups in terms of perceived nation-level tightness–looseness and general tightness–looseness of life. The scores of the high-tightness perception group were higher than those of the low-tightness perception group for each CTLS and GTLS item, and there was no significant difference in the gender and age compositions of the two groups. This indicates that the CTLS and GTLS have good structural validity and can distinguish individuals with different perceived tightness–looseness preferences. When compared with Western countries, Chinese culture is tighter (Uz, 2015) yet the perception of cultural tightness–looseness remains heterogeneous at a personal level. Consistent with these findings, previous work using the LPA method demonstrates that, although China is a typical collectivist country, it can be divided into two subgroups dominated by individualism and collectivism at the individual level (O'Neill et al., 2016).

In addition, the reliability test showed that the revised CTLS and GTLS had acceptable internal consistency reliability (*α* > 0.7, Viladrich et al., 2017), suggesting that each item in CTLS and GTLS effectively measures the same construct. Pearson’s correlation and intraclass correlation were used to examine test–retest reliability. Although the Pearson correlation coefficient was less than the 0.8 criteria (Porst et al., 2007), ICC indicated that the revised CTLS and GTLS reached acceptable retest reliability (ICC > 0.4, Fleiss et al., 2013). Therefore, the revised CTLS and GTLS showed acceptable consistency and stability.

The revised Chinese versions of the CTLS and GTLS have good criterion validity. Both CTLS and GTLS scores positively correlated with dutifulness, impulse control, and personal structural needs. Previous studies have found that both CTLS and GTLS are related to dutifulness because people are concerned with compliance with norms and fulfilling prescribed responsibilities and obligations in tightness culture (Gelfand et al., 2011; Harrington, 2017). People in tightness culture need to adjust their behaviors to conform to social norms, so they have high self-control (Gelfand et al., 2011; Mu et al., 2015). In addition, individuals with high simple structure desire prefer a tightly organized life (Neuberg and Newsom, 1993), so there was a positive association between personal structural needs and perceived tightness–looseness as found by predecessors (Gelfand et al., 2011; Harrington, 2017). Similar to previous findings, this study found that perceived cultural tightness was positively associated with life satisfaction and positive emotional experience (Chua et al., 2019). In addition, Liu et al. (2022) found that perceived social norms can reduce negative emotional experiences and enhance confidence in fighting COVID-19 in the Chinese population. Therefore, greater cultural tightness is likely to result in beneficial psychological outcomes in the Chinese context. The findings of this study can enrich the downstream factor of cultural tightness theory, that is, the influence of cultural tightness on psychological well-being.

Cultural tightness and looseness play an important role in responding to a variety of threats (Gelfand et al., 2021). For example, countries with greater cultural tightness have fewer confirmed COVID-19 cases and deaths (Gelfand et al., 2021). At the individual level, perceived higher cultural tightness results in more support for policies to help control COVID-19, more public health behaviors (e.g., wearing masks and handwashing) (Gilliam et al., 2022) and lower levels of COVID-19 risk perception (Liu et al., 2022). Conversely, epidemics may change the tightness–looseness degree of a culture (Gelfand et al., 2011). Whether cultural tightness–looseness is affected by COVID-19 is worth comparing in future studies. In addition, tightness culture is associated with greater prejudice against other races, religions, sexual orientations and immigrants, which may lead to intercultural conflict (Jackson et al., 2019). Therefore, we should also pay attention to the inhibition role of cultural tightness in the process of sociocultural change.

### 4.1. Limitations and future directions

Harrington (2017) developed several domains of the tightness–looseness scale whereas this study only adapted the GTLS. The applicability of other scales to a Chinese population requires further research. Moreover, only demographic information on gender and age was collected in this study to protect the privacy of participants. Future studies should consider collecting other demographic variables, such as participants’ education level, socio-economic status, and living area, to better understand the variation of perceived cultural tightness among different sub-groups. Finally, further research should expand the age range of the sample to test the reliability and validity of the CTLS-Chinese and GTLS-Chinese amongst different age groups, such as older adults and children.

## 5. Conclusion

In conclusion, cultural tightness and looseness have an important impact on people’s behavior and psychology. The CTLS and GTLS scales adapted in this study can provide reliable and effective measures of individuals’ perceived tightness–looseness in their national culture and general life in the Chinese context.

## Data availability statement

The datasets presented in this study can be found in online repositories. The names of the repository/repositories and accession number(s) can be found at: https://osf.io/zprsy/files/osfstorage.

## Ethics statement

The studies involving human participants were reviewed and approved by Ethics Committee of the Department of Psychology, Renmin University of China. Written informed consent to participate in this study was provided by the participants’ legal guardian/next of kin.

## Author contributions

JL collected and analyzed the data, and wrote and revised the manuscript. HM collected the data and helped revise the manuscript. XL helped analyze the data and revise the manuscript. PH designed the work, provided data analysis advice, and revised the manuscript. All authors contributed to the article and approved the submitted version.

## Funding

This study was supported by National Social Science Foundation of China (Major Program) (19ZDA021) and by Fund for building world-class universities (disciplines) of Renmin University of China.

## Conflict of interest

The authors declare that the research was conducted in the absence of any commercial or financial relationships that could be construed as a potential conflict of interest.

## Publisher’s note

All claims expressed in this article are solely those of the authors and do not necessarily represent those of their affiliated organizations, or those of the publisher, the editors and the reviewers. Any product that may be evaluated in this article, or claim that may be made by its manufacturer, is not guaranteed or endorsed by the publisher.

## References

[ref1] Akin A. Hamedoglu M. A. Kaya Ç. Sarıçam H. (2013). Turkish version of the work and meaning inventory (WAMI): validity and reliability study. J. Eur. Educ. 3, 11–16.

[ref2] Baek J. Kim K. A. Kim H. Kim O. Ko M. Kim S. H.. (2022). The validity of ICD-11 PTSD and complex PTSD in north Korean defectors using the international trauma questionnaire. Eur. J. Psychotraumatol. 13:2119012. doi: 10.1080/20008066.2022.2119012 36237828 PMC9553178

[ref3] Baumgartner H. Weijters B. Pieters R. (2018). Misresponse to survey questions: a conceptual framework and empirical test of the effects of reversals, negations, and polar opposite core concepts. J. Mark. Res. 55, 869–883. doi: 10.1177/0022243718811848

[ref4] Bronfenbrenner U. (1992). Ecological systems theory. London Jessica Kingsley Publishers.

[ref5] Campbell A. (1976). Subjective measures of well-being. Am. Psychol. 31, 117–124. doi: 10.1037/0003-066X.31.2.117, PMID: 1267244

[ref6] Carpenter S. (2000). Effects of cultural tightness and collectivism on self-concept and causal attributions. Cross-Cult. Res. 34, 38–56. doi: 10.1177/106939710003400103

[ref7] Chan D. K. S. (1996). Tightness-looseness revisited: some preliminary analyses in Japan and the United States. Int. J. Psychol. 31, 1–12. doi: 10.1080/002075996401179

[ref8] Chen Y. Huang Y. H. Wang L. Shi J. Q. (2008). The revision of the personal need for structure scale in Chinese. Acta Sci. Nat. Univ. Pekin. 44, 490–492. doi: 10.3321/j.issn:0479-8023.2008.03.025

[ref9] Chua R. Y. Huang K. G. Jin M. (2019). Mapping cultural tightness and its links to innovation, urbanization, and happiness across 31 provinces in China. Proc. Natl. Acad. Sci. 116, 6720–6725. doi: 10.1073/pnas.1815723116, PMID: 30833399 PMC6452675

[ref10] Davis D. E. Rice K. Hook J. N. Van Tongeren D. R. DeBlaere C. Choe E.. (2015). Development of the sources of spirituality scale. J. Couns. Psychol. 62, 503–513. doi: 10.1037/cou0000082, PMID: 26010288

[ref11] Du J. Wang Y. Xu S. Huang Y. Zhang R. Xiao L.. (2022). Structural model of napping motivation among Chinese college students based on self-rating: evidence from an exploratory factor analysis. Nat. Sci. Sleep 14, 843–853. doi: 10.2147/NSS.S349013, PMID: 35529049 PMC9075905

[ref12] Ferguson S. L. Moore E. W. G. Hull D. M. (2020). Finding latent groups in observed data: a primer on latent profile analysis in Mplus for applied researchers. Int. J. Behav. Dev. 44, 458–468. doi: 10.1177/0165025419881721

[ref13] Fleiss J. L. Levin B. Paik M. C. (2013). Statistical methods for rates and proportions. New York, NY John Wiley & Sons.

[ref14] Ge P. (2016). The revision of the big five personality inventory (IPP-NEO-120) Master dissertation Yangzhou Yangzhou University

[ref15] Gedik Y. Ozbek M. F. (2020). How cultural tightness relates to creativity in work teams: exploring the moderating and mediating mechanisms. Creat. Innov. Manag. 29, 634–647. doi: 10.1111/caim.12409

[ref16] Gelfand M. J. Jackson J. C. Pan X. Nau D. Pieper D. Denison E.. (2021). The relationship between cultural tightness–looseness and COVID-19 cases and deaths: a global analysis. Lancet Planet Health 5, e135–e144. doi: 10.1016/S2542-5196(20)30301-6, PMID: 33524310 PMC7946418

[ref17] Gelfand M. J. Nishii L. H. Raver J. L. (2006). On the nature and importance of cultural tightness-looseness. J. Appl. Psychol. 91, 1225–1244. doi: 10.1037/0021-9010.91.6.1225, PMID: 17100480

[ref18] Gelfand M. J. Raver J. L. Nishii L. Leslie L. M. Lun J. Lim B. C.. (2011). Differences between tight and loose cultures: a 33-nation study. Science 332, 1100–1104. doi: 10.1126/science.1197754, PMID: 21617077

[ref19] Gilliam A. Schwartz D. B. Godoy R. Boduroglu A. Gutchess A. (2022). Does state tightness-looseness predict behavior and attitudes early in the COVID-19 pandemic in the USA? J. Cross-Cult. Psychol. 53, 522–542. doi: 10.1177/00220221221077710

[ref20] Gómez-López M. Chicau Borrego C. Marques da Silva C. Granero-Gallegos A. González-Hernández J. (2020). Effects of motivational climate on fear of failure and anxiety in teen handball players. Int. J. Environ. Res. Public Health 17:592. doi: 10.3390/ijerph17020592, PMID: 31963331 PMC7013665

[ref21] Gow M. (2017). The core socialist values of the Chinese dream: towards a Chinese integral state. Crit. Asian Stud. 49, 92–116. doi: 10.1080/14672715.2016.1263803

[ref22] Harrington J. R. (2017). Worlds unto themselves: Tightness-looseness and social class Doctoral dissertation University of Maryland College Park.

[ref23] Harrington J. R. Gelfand M. J. (2014). Tightness–looseness across the 50 United States. Proc. Natl. Acad. Sci. 111, 7990–7995. doi: 10.1073/pnas.1317937111, PMID: 24843116 PMC4050535

[ref24] Hawkins R. X. Goodman N. D. Goldstone R. L. (2019). The emergence of social norms and conventions. Trends Cogn. Sci. 23, 158–169. doi: 10.1016/j.tics.2018.11.003, PMID: 30522867

[ref25] He S. Qian J. (2017). From an emerging market to a multifaceted urban society: urban China studies. Urban Stud. 54, 827–846. doi: 10.1177/0042098016675826

[ref26] Howard M. C. Hoffman M. E. (2018). Variable-centered, person-centered, and person-specific approaches: where theory meets the method. Organ. Res. Methods 21, 846–876. doi: 10.1177/1094428117744021

[ref27] Jackson J. C. Gelfand M. De S. Fox A. (2019). The loosening of American culture over 200 years is associated with a creativity–order trade-off. Nat. Hum. Behav. 3, 244–250. doi: 10.1038/s41562-018-0516-z 30953010

[ref28] Jackson J. C. Van Egmond M. Choi V. K. Ember C. R. Halberstadt J. Balanovic J.. (2019). Ecological and cultural factors underlying the global distribution of prejudice. PLoS One 14:e0221953. doi: 10.1371/journal.pone.0221953 31490981 PMC6730889

[ref29] Johnson J. A. (2014). Measuring thirty facets of the five factor model with a 120-item public domain inventory: development of the IPIP-NEO-120. J. Res. Pers. 51, 78–89. doi: 10.1016/j.jrp.2014.05.003

[ref30] Liu P. Chan D. Qiu L. Tov W. Tong V. J. C. (2018). Effects of cultural tightness–looseness and social network density on expression of positive and negative emotions: a large-scale study of impression management by Facebook users. Personal. Soc. Psychol. Bull. 44, 1567–1581. doi: 10.1177/0146167218770999, PMID: 29742997

[ref31] Liu H. H. Peng F. Zeng X. H. Zhao J. B. Zhang X. Y. (2019). Authoritarian personality and subjective well-being in Chinese college students: the moderation effect of the organizational culture context. Personal. Individ. Differ. 138, 79–83. doi: 10.1016/j.paid.2018.09.030

[ref32] Liu S. Zhu J. Liu Y. Wilbanks D. Jackson J. C. Mu Y. (2022). Perception of strong social norms during the COVID-19 pandemic is linked to positive psychological outcomes. BMC Public Health 22, 1–12. doi: 10.1186/s12889-022-13744-2 35869459 PMC9305059

[ref33] Ma A. Savani K. Liu F. Tai K. Kay A. C. (2022). The mutual constitution of culture and psyche: the bidirectional relationship between individuals’ perceived control and cultural tightness–looseness. J. Person. Soc. Psychol. Online ahead of print. doi: 10.1037/pspa0000327, PMID: 36315622

[ref34] Mandel A. Realo A. (2015). Across-time change and variation in cultural tightness-looseness. PLoS One 10:e0145213. doi: 10.1371/journal.pone.0145213, PMID: 26683813 PMC4684201

[ref35] Marcus J. Aksoy E. Tesfa Alemu G. (2022). Perceptions of organizational tightness–looseness moderate associations between perceived unfair discrimination and employees’ job attitudes. J. Cross Cult. Psychol. 53, 426–445. doi: 10.1177/00220221221077376

[ref36] Mu Y. Kitayama S. Han S. Gelfand M. J. (2015). How culture gets embrained: cultural differences in event-related potentials of social norm violations. Proc. Natl. Acad. Sci. 112, 15348–15353. doi: 10.1073/pnas.1509839112, PMID: 26621713 PMC4687606

[ref37] Neuberg S. L. Newsom J. T. (1993). Personal need for structure: individual differences in the desire for simpler structure. J. Pers. Soc. Psychol. 65, 113–131. doi: 10.1037/0022-3514.65.1.113

[ref38] Oh S. (2022). Cultural tightness, neuroticism, belief in a just world for self, gender, and subjective well-being: a moderated mediation model. Curr. Psychol. 41, 8300–8311. doi: 10.1007/s12144-022-03652-4

[ref39] O'Neill T. A. McLarnon M. J. Xiu L. Law S. J. (2016). Core self-evaluations, perceptions of group potency, and job performance: the moderating role of individualism and collectivism cultural profiles. J. Occup. Organ. Psychol. 89, 447–473. doi: 10.1111/joop.12135

[ref40] Paschke K. Austermann M. I. Thomasius R. (2020). Assessing ICD-11 gaming disorder in adolescent gamers: development and validation of the gaming disorder scale for adolescents (GADIS-A). J. Clin. Med. 9:993. doi: 10.3390/jcm9040993, PMID: 32252305 PMC7230491

[ref41] Porst H. Gilbert C. Collins S. Huang X. Symonds T. Stecher V.. (2007). Development and validation of the quality of erection questionnaire. J. Sex. Med. 4, 372–381. doi: 10.1111/j.1743-6109.2006.00422.x, PMID: 17367432

[ref42] Raylu N. Oei T. P. (2004). The Gambling Related Cognitions Scale (GRCS): development, confirmatory factor validation and psychometric properties. Addiction 99, 757–769. doi: 10.1111/j.1360-0443.2004.00753.x, PMID: 15139874

[ref43] Siemens J. C. Raymond M. A. Choi Y. Choi J. (2020). The influence of message appeal, social norms and donation social context on charitable giving: investigating the role of cultural tightness-looseness. J. Mark. Theory Pract. 28, 187–195. doi: 10.1080/10696679.2020.1717968

[ref44] Suh Y. (2015). The performance of maximum likelihood and weighted least square mean and variance adjusted estimators in testing differential item functioning with nonnormal trait distributions. Struct. Equ. Model. Multidiscip. J. 22, 568–580. doi: 10.1080/10705511.2014.937669

[ref45] Tein J. Y. Coxe S. Cham H. (2013). Statistical power to detect the correct number of classes in latent profile analysis. Struct. Equ. Model. Multidiscip. J. 20, 640–657. doi: 10.1080/10705511.2013.824781, PMID: 24489457 PMC3904803

[ref46] Treviño L. J. Egri C. P. Ralston D. A. Naoumova I. Furrer O. Li Y.. (2021). A multi-country, multi-sector replication challenge to the validity of the cultural tightness-looseness measure. Asia Pac. J. Manag. 38, 735–764. doi: 10.1007/s10490-019-09682-0

[ref47] Uz I. (2015). The index of cultural tightness and looseness among 68 countries. J. Cross-Cult. Psychol. 46, 319–335. doi: 10.1177/0022022114563611

[ref48] Viladrich C. Angulo-Brunet A. Doval E. (2017). A journey around alpha and omega to estimate internal consistency reliability. Ann. Psychol. 33, 755–782. doi: 10.6018/analesps.33.3.268401

[ref49] Wang X. Wang X. Ma H. (1999). The handbook of mental health assessment among Chinese. Beijing: Chinese Mental Health Journal Press

[ref50] Yildiz E. Akpinar E. Tatar N. Ergin O. (2009). Exploratory and confirmatory factor analysis of the metacognition scale for primary school students. Educat. Sci. 9, 1591–1604.

[ref51] Youn N. Park J. Eom H. J. (2019). Reactions to nonconformity imagery in advertising among Chinese and Japanese consumers: the effect of personal and national cultural tightness. J. Advert. 48, 532–554. doi: 10.1080/00913367.2019.1674754

