# Supplementary material for: Validation of the Chinese Cultural Tightness–Looseness Scale and General Tightness–Looseness Scale
Source: Front Psychol. 2023 Apr 18;14:1131868. doi: 10.3389/fpsyg.2023.1131868 (PMC10153570; doi:10.3389/fpsyg.2023.1131868)
Supplement: Supplementary file 1 [file Data_Sheet_1.pdf]

English and Chinese version of CTLS and GTLS

CTLS-revised(修订后文化松紧量表）

Instruction: The following statements refer to your country as a whole. Please indicate the degree that you agree with the following statements using the following 6-point scale (1=strongly disagree, 6=strongly agree). Note that the "social norms" appeared in these statements are standards for behavior that are generally unwritten.

指导语：以下是一些对国家整体的描述。请根据您所了解的真实情况对以下描述做出评价。请注意，“社会规范”在下列说法中是指一些没有被明文规定的社会行为标准。1=非常不同意，6=非常同意

Items	题目
CTLS1 There are many social norms that people are supposed to abide by in this country.	1. 在这个国家，有很多社会规范需要遵守。
CTLS2 In this country, there are very clear expectations for how people should act in most situations.	2. 在这个国家，大多数情况下人们很清楚应该如何作为。
CTLS3 People agree upon what behaviors are appropriate versus inappropriate in most situations this country.	3. 在这个国家，大多数情况下大家对什么是妥当或者不妥当的行为有很大程度的共识。
CTLS4 People in this country have a great deal of freedom in deciding how they want to behave in most situations.	4.大多数情况下人们可以充分地自由决定作为。
CTLS5 In this country, if someone acts in an inappropriate way, others will strongly disapprove.	5. 在这个国家，如果有人在做出不妥的违规行为会受到来自其他人的强烈的反对。
CTLS6 People in this country almost always comply with social norms.	6. 在这个国家，人们几乎总是会遵守社会规范。

GTLS-revised 一般生活松紧量表（修订后）

Instruction: The following statements refer to your life as a whole. Please indicate the degree that you agree with the following statements using the following 6-point scale (1=strongly disagree, 6=strongly agree).

指导语：你的生活是一个整体，请用下列量表说明你是否同意下列陈述。1=非常不同意，6=非常同意

Items	题目
GTLS1 There are many rules that I am supposed to follow in my life.	1. 在我的生活中有很多我应该遵守的规则。
GTLS2 In my life , there are very clear expectations for how I should act in most situation.	2. 在我的生活中，我在大多数情况下应该如何行动都有非常明确的期望。
GTLS3 It is clear what behaviors are appropriate versus inappropriate in my life.	3. 在我的生活中，什么行为是合适的，什么行为是不合适的，有很清楚的标准。
GTLS4 I have a great deal of freedom in deciding how I want to behave in most situations.	4. 在我的生活中，大多数情况下我可以充分地自由决定如何表现。
GTLS5 In my life, if I act in an inappropriate way, others will strongly disapprove.	5. 在我的生活中，如果我的行为不当，别人会强烈反对。
GTLS6 In my life, I almost always follow the rules.	6. 我几乎总是遵守规则。
GTLS7 In my life, people closely monitor what I do.	7. 在我的生活中，人们密切关注着我所做的事情。
GTLS8 In my life, there are strong punishments if I don’t follow the rules.	8. 在我的生活中，如果我不遵守规则，就会受到严厉的惩罚。
GTLS9 My life is very structured. I know what I should and should not be doing.	9. 我的生活很有条理， 我知道我该做什么，不该做什么。

GTLS10 In my life, there is a right way and a wrong way to do things.	10. 在我的生活中，做事的方法被分为正确的和错误的。
GTLS11 There is a rule or a proper procedure for most things.	11. 大多数事情都有一个规则或一个适当的程序。
GTLS12 I often have a choice in deciding what I want to do in my life.	12. 我经常可以选择我想要的生活。
GTLS13 I often have a choice in deciding when I want to do something in my life.	13. 在决定我想做的事情的时候，我经常可以选择。

Scree plot of EFA

Figure S1. The Scree plot of EFA for CTLS (four items).

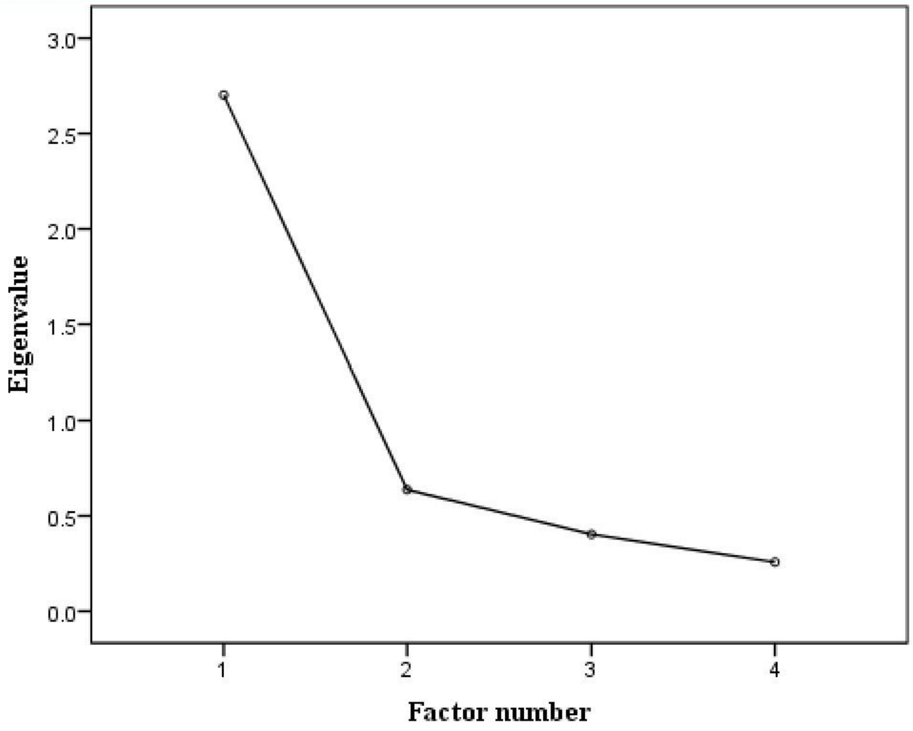

Figure S2. The Scree plot of EFA for GTLS (eight items).

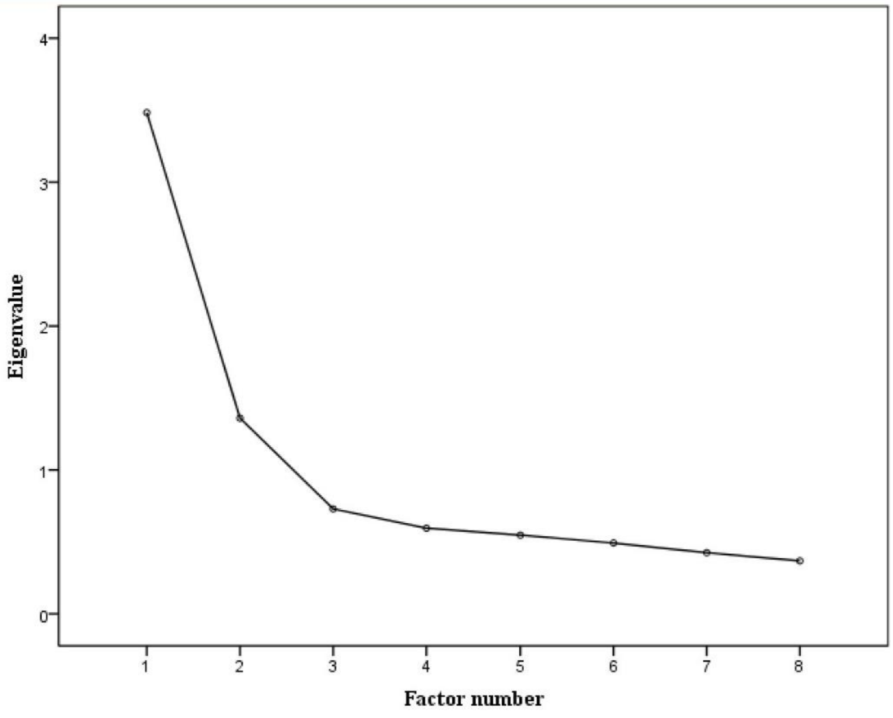

LPA supplementary analysis

Sometimes, each model fitting index may not show consistency. For example, when 3-class were reserved, LMR was not significant and 2-class was supported, while BLRT was significant and 3 or more classes was supported. Statistical indicators only provide reference for researchers to make decisions. We should also consider the interpretation of classes when determining the best model. In this study, 1-class model was excluded because it yielded higher AIC, BIC, and aBIC values than other models. 4-class model was excluded because it had lower entropy and higher BIC than the 3-class model, and the LMR test was not significant. In addition, we plotted the mean values from 3-class solution (see figure S3). We did not find that there exist profiles such as high-compliance-low-sanction and low-compliance-high-sanction in the GTLS scores. What's more, the LMR of 3-class compared with 2-class was not significant. We thought that a more concise model (2-class) would show that the revised GTLS can distinguish differences in participants perceived general life tightness, with a classification accuracy greater than 90% (entropy = 0.88). Therefore, 2-class model was retained.

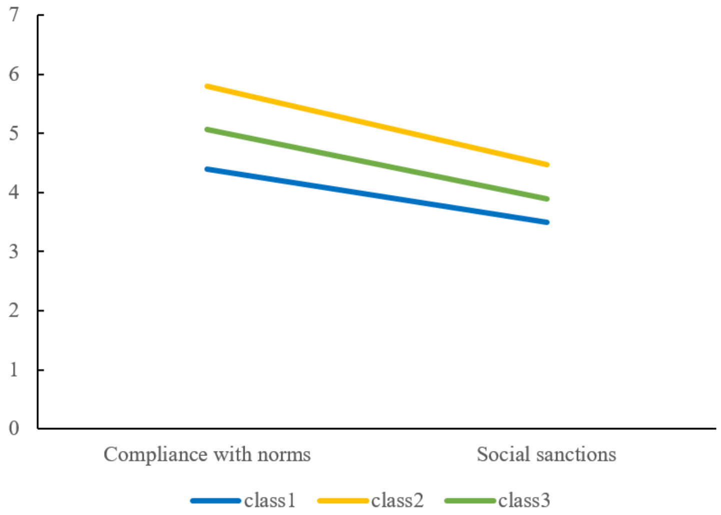

Figure S3. Mean scores on compliance with norms and social sanctions of 3-class LPA.

Criterion Validity supplementary analysis

Table S1 SEM with compliance with social norms and social sanctions as predictors simultaneously

Path	Estimate	Path	Estimate
Compliance with norms		Social sanctions	
→ Dutifulness	0.78***	→ Dutifulness	-0.12*
→Impulse control	0.56***	→Impulse control	0.04
→Personal need for structure	0.54***	→Personal need for structure	0.05
→Well-Being	0.78***	→Well-Being	0.18

We constructed a SEM with compliance with social norms and social sanctions as predictors (they were correlate with each other), and dutifulness, impulse control, personal need for structure, and well-being as dependent variables simultaneously. Results showed that the model fitted the data well (χ^2 /df = 2.21, CFI = 0.91, TLI = 0.90, RMSEA = 0.05). However, only compliance with social norms positively predicted these criterion variables. The prediction of social sanctions on criterion variables was not significant or even reversed. We conducted a multicollinearity test and found that there was a moderate multicollinearity (eigenvalue < 0.05 and the 15 < condition index < 30; Shrestha, 2020) when compliance with norms and social sanctions were included simultaneously in a linear regression model.

The multicollinearity may lead to large changes in regression coefficient estimates.

Reference

Shrestha, N. (2020). Detecting multicollinearity in regression analysis. *American Journal of Applied Mathematics and Statistics, 8*(2), 39-42.
